# Synthesis and crystallographic characterization of a mononuclear cobalt(III) complex possessing both thiol­ate and thio­ether donors: reactivity of an thiol­ate-bridged penta­nuclear Co_2_Ag_3_ complex with iodo­methane

**DOI:** 10.1107/S2056989017005229

**Published:** 2017-04-11

**Authors:** Yosuke Fukuda, Nobuto Yoshinari, Takumi Konno

**Affiliations:** a Department of Chemistry, Graduate School of Science, Osaka University, Toyonaka, Osaka 560-0043, Japan

**Keywords:** crystal structure, alkyl­ation reaction, coordination compound, thiol­ate complex, thio­ether complex, cobalt ion

## Abstract

Treatment of an S-bridged penta­nuclear Ag^I^_3_Co^III^_2_ complex, [Ag_3_{Co(*L*)}_2_]^3+^ [*L* ^3–^ = N(CH_2_NHCH_2_CH_2_S^−^)_3_], in which two tris­(thiol­ate)-type mononuclear Co^III^ units ([Co(*L*)]) are bridged by three Ag^I^ ions through S atoms, with iodo­methane (CH_3_I) gave a new Co^III^ mononuclear complex, [Co(*L*Me_2_)]^2+^ [*L*Me_2_^−^ = N(CH_2_NHCH_2_CH_2_S^−^)(CH_2_NHCH_2_CH_2_SCH_3_)_2_], systematic name: {2-[(bis{[2-(methylsulfanyl)ethyl]aminomethyl}aminomethyl)amino]ethanethiolato}cobalt(III) bis(hexafluoridophosphate). This cationic complex was crystallized with PF_6_^−^ anions to form the title compound, [Co(*L*Me_2_)](PF_6_)_2_. In the [Co(*L*Me_2_)]^2+^ cation, two of three thiol­ate groups in [Co(*L*)] are methyl­ated while one thiol­ate group remains unreacted. Although a total of eight stereoisomers are possible for [Co(*L*Me_2_)]^2+^, only a pair of enanti­omers {Λ_*RR*_- and Δ_*SS*_-[Co(*L*Me_2_)]^2+^} are selectively formed. In the crystal, the complex cations and the PF­_6_^−^ anions are connected through weak N—H⋯F, C—H⋯F and C—H⋯S hydrogen bonds into a three-dimensional structure. Two F atoms in one PF_6_ anion are disordered over two sets of sites with refined occupancies of 0.61 (4) and 0.39 (4) and two F atoms in the other PF_6_^−^ anion are disordered over two sets of sites with occupancies of 0.5.

## Abstract 2

A new mononuclear cobalt(III) complex that has two thiol­ate and one thio­ether donor atoms is reported.

## Chemical context   

It has long been recognized that thiol­ate groups (*R* ^1^S^−^) bound to a transition metal center readily react with alkyl halides (*R* ^2^ *X*) to form a transition metal complex with thio­ether groups (*R* ^1^ *SR* ^2^). Since the resulting thio­ether S atoms generally turn to be asymmetric (chiral), the alkyl­ated species are an inter­esting research target of coordination stereochemistry. Among a variety of alkyl halides, iodo­methane (CH_3_I) is one of the most common alkyl­ation reagent because of its high reactivity and simple mol­ecular structure. For example, the reaction of a mono(thiol­ate)-type Co^III^ mononuclear complex, [Co(aet)(en)_2_]^2+^ (aet = NH_2_CH_2_CH_2_S^−^, en = ethyl­enedi­amine), with iodo­methane selectively produces the corresponding mono(thio­ether)-type complex, [Co(mtea)(en)_2_]^3+^ (mtea = NH_2_CH_2_CH_2_SCH_3_) (Elder *et al.*, 1978 ▸). Moreover, Busch *et al.* (1964 ▸) showed that a bis­(thiol­ate)-type Ni^II^ complex, [Ni(aet)_2_], is also easily converted to the corres­ponding bis­(thio­ether)-type complex, [Ni(mtea)_2_]^2+^, by treating with iodo­methane. Unlike mono(thiol­ate)- or bis­(thiol­ate)-type complexes, tris­(thiol­ate)-type complexes have been found to show different reactivity toward iodo­methane. That is, the reaction of a tris­(thiol­ate)-type mononuclear rhodium(III) complex, *fac*(*S*)-[Rh(aet)_3_], with iodo­methane afforded a unique di­methyl­ated mono(thiol­ate)bis­(thio­ether)-type complex, *fac*(*S*)-[Rh(aet)(mtea)_2_]^2+^, whereas the mono­methyl­ated bis­(thiol­ate)mono(thio­ether)-type and tri­methyl­ated tris­(thio­ether)-type species were little formed (Hirotsu *et al.*, 2002 ▸). Based on the ^13^C{^1^H} NMR measurements, it was suggested that only a pair of enanti­omers is formed for *fac*(*S*)-[Rh(aet)(mtea)_2_]^2+^. However, the lack of crystallographic analytical data for *fac*(*S*)-[Rh(aet)(mtea)_2_]^2+^ prevented the further study on the stereochemistry of the di­alkyl­ated complex.
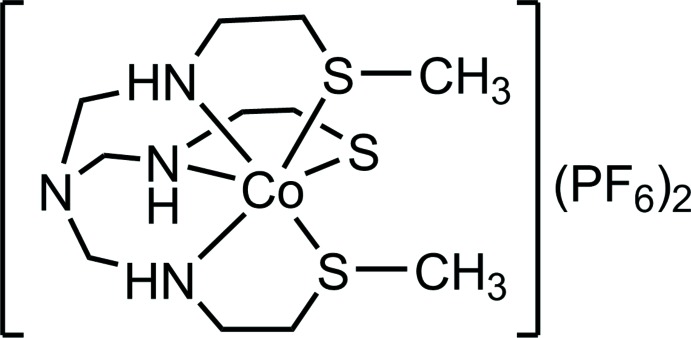


In the course of our continuing study of the alkyl­ation reaction of metal complexes with amino­thiol­ate ligands (Okamoto *et al.*, 1999 ▸; Chikamoto *et al.*, 2005 ▸, 2007 ▸; Yoshinari & Konno, 2008 ▸, 2009 ▸), we herein report that an S-bridged Ag^I^_3_Co^III^_2_ penta­nuclear complex, {Ag_3_[Co(*L*)]_2_}^3+^ [*L* ^3–^ = N(CH_2_NHCH_2_CH_2_S^−^)_3_] (Tokuda *et al.*, 2000 ▸), in which two tris­(thiol­ate)-type octa­hedrally shaped Co^III^ moieties with an aet derivative ligand, [Co(*L*)], are linearly linked by three Ag^I^ ions, reacts with iodo­methane to give a mono(thiol­ate)bis­(thio­ether)-type complex, [Co(*L*Me_2_)]^2+^ [*L*Me_2_^−^ = N(CH_2_NHCH_2_CH_2_S^−^)(CH_2_NHCH_2_CH_2_SCH_3_)_2_]. It is noteworthy that the complex was crystallized as a hexa­fluorido­phosphate salt, [Co(*L*Me_2_)](PF_6_)_2_, and its mol­ecular structure was fully determined by single-crystal X-ray diffraction analysis. As far as we know, this is the first crystallographic characterization of a cobalt(III) complex that has two thio­ether and one thiol­ate donor groups. In addition, this is a unique example of a direct conversion of a thiol­ate-bridged multinuclear complex to a mononuclear thio­ether complex by alkyl­ation reaction. Treatment of the thiol­ate-bridged penta­nuclear complex {Ag_3_[Co(*L*)]_2_}^3+^ with excess iodo­methane in water gave a greenish-brown suspension. After removing the insoluble solid by filtration, the purple–brown filtrate was purified by a cation–exchange column (SP-Sephadex C-25). The product was isolated as purple–brown crystals by adding a hexa­fluorido­phosphate anion. The geometrical parameters and stereoisomerism of the title compound based on the X-ray analysis, together with the spectroscopic data, are described in this paper.

## Structural commentary   

**Figure 1:**
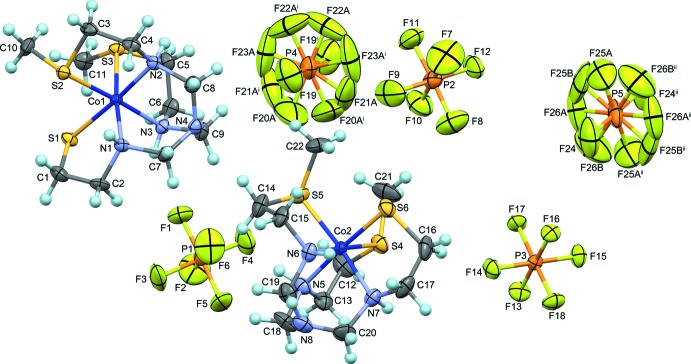
A perspective view of the molecular components in the title compound with the atom-numbering scheme. Displacement ellipsoids are drawn at the 50% probability level. H atoms are shown as light-blue balls. [Symmetry codes: (i) −*x*, *y*, −*z* + 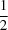; (ii) −*x* + 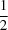, −*y* + 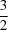, −*z*.]

X-ray structural analysis revealed that there are two crystallographically independent yet essentially the same complex cations, [Co(*L*Me_2_)]^2+^, and four PF_6_^−^ anions in the asymmetric unit (Fig. 1 ▸). The number of PF_6_^−^ anions indicates that each complex cation is divalent. Each complex cation consists of a hexa­dentate-*N*,*N*′,*N*′′,*S*,*S*′,*S*′′-binding *L*Me_2_^−^ ligand that coordinates to a Co^III^ atom in a slightly distorted octa­hedral geometry. This result clearly indicates that two of three thiol­ate groups in the [Co(*L*)] moiety were methyl­ated to form [Co(*L*Me_2_)]^2+^. No apparent difference was observed among the Co—S bond lengths for thiol­ate S atoms (S_thiol­ate_) [2.2384 (13)–2.2478 (11) Å] and those for thio­ether S atoms (S_thio­ether_) [2.2190 (13)–2.2599 (11) Å] in [Co(*L*Me_2_)]^2+^. However, the Co—N bonds *trans* to S_thiol­ate_ [2.061 (4)–2.062 (3) Å] are *ca* 0.05 Å longer than the Co—N bonds *trans* to S_thio­ether_ [2.004 (4)–2.020 (4) Å]. The difference is reasonably explained by the decrease of the *trans* influence due to the alkyl­ation on S atoms. As a result of the steric repulsion between the methyl groups on the S atoms, the S—Co—S angles in [Co(*L*Me_2_)]^2+^ deviate considerably from 90° [86.58 (4)–95.07 (4)°].

**Figure 2:**
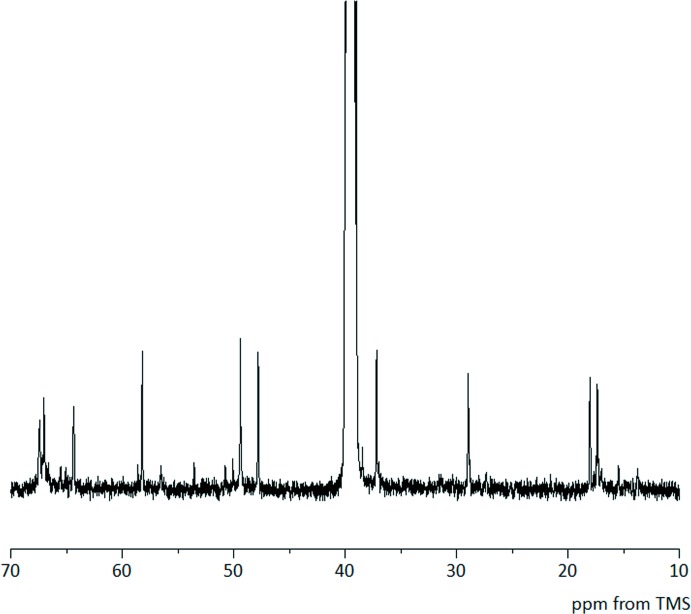
^13^C{H} NMR spectrum of the title compound in DMSO-*d*_6_.

Each Co^III^ ion is surrounded by three S and three N atoms in a *fac-*(*S*) geometry, like the parent [Co(*L*)] units. Considering the absolute configurations of the cobalt(III) atom (Δ and Λ) and the two asymmetric sulfur atoms (*R* and *S*), four pairs of diastereomers, Δ_*SS*_/Λ_*RR*_, Δ_*SR*_/Λ_*RS*_, Δ_*RS*_/Λ_*SR*_ and Δ_*RR*_/Λ_*SS*_, are possible for [Co(*L*Me_2_)]^2+^. However, the asymmetric unit of this crystal contains two Λ_*RR*_ isomers. As indicated by the space group *C*2/*c*, the title crystal is a racemic compound consisting of a pair of enanti­omers, Λ_*RR*_ and Δ_*SS*_. This result is consistent with the observation that the ^13^C{^1^H} NMR spectrum of the title compound in DMSO-*d*_6_ exhibits a total of 10 sharp singlet signals, assignable to the *C*_1_ symmetrical Λ_*RR*_ and Δ_*SS*_ isomers of [Co(LMe_2_)]^2+^ (Fig. 2 ▸). For both complex cations [Co(*L*Me_2_)]^2+^ in the crystal, two of three *N*,*S*-chelate rings have a *gauche* form with the *lel* (λ for Δ and δ for Λ) conformation, while one has a *gauche* form with the *ob* (λ for Λ and δ for Δ) conformation.

In summary, we report here the first example of a crystallographically characterized mono(thiol­ate)bis­(thio­ether)-type mononuclear cobalt(III) complex, [Co(*L*Me_2_)]^2+^. This complex was obtained by the unprecedented direct conversion of a thiol­ate-bridged Ag^I^_3_Co^III^_2_ penta­nuclear complex by alkyl­ation reaction using iodo­methane. The selective formation of the Λ_*RR*_ and Δ_*SS*_ isomers of [Co(*L*Me_2_)]^2+^ observed in the crystal structure is consistent with the result of ^13^C{^1^H} NMR. The findings reported herein will provide insight into the synthesis and structures of coordination compounds containing both thiol­ate and thio­ether donor groups.

## Supra­molecular features   

In the crystal, the complex cations and the PF_6_^−^ anions are connected through many weak N—H⋯F, C—H⋯F and C—H⋯S hydrogen bonds (Table 1 ▸), forming a three-dimensional structure.

## Synthesis and crystallization   

To a dark-purple solution of {Ag_3_[Co(*L*)]_2_}(NO_3_)_3_·4H_2_O (0.30 g, 0.25 mmol) in 100 ml of water was added CH_3_I (0.5 ml, 8.0 mmol). The mixture was stirred at room temperature for 1.5 days in the dark. After removing a brown powder (200 mg) by filtration, the purple–brown filtrate was poured onto an SP-Sephadex C-25 column (Na^+^ form, 1.5 × 30 cm). First, a purple band was eluted with 0.05 *M* aqueous NaCl. Then, a purple–brown band of [Co(*L*Me_2_)]^2+^ was eluted with 0.15 *M* aqueous NaCl. To the concentrated purple–brown eluate was added 1.0 *M* aqueous NH_4_PF_6_ (5 ml) and the solution was allowed to stand at room temperature for 20 d. The resulting dark purple–brown block crystals of the title compound were collected by filtration. Yield: 0.08 g (29%). Single crystals suitable for X-ray analysis were obtained by recrystallization from water by adding 1.0 *M* aqueous NH_4_PF_6_. Analysis: calculated for [Co(*L*Me_2_)](PF_6_)_2_: C 20.01, H 4.12, N 8.48%; found: C 20.25, H 4.06, N 8.51%. ^13^C{^1^H} NMR (DMSO-*d*_6_): δ 17.40, 18.05, 28.97, 37.20, 47.82, 49.42, 58.22, 64.39, 67.05, 67.50. One of the ^13^C signals overlaps with the signal from solvent. IR(KBr, ν cm^−1^): 3266.8(*m*), 3029.6(*w*), 1432.8(*m*), 1245.8(*w*), 1158.0(*w*), 1113.7(*w*), 1034.6(*w*), 955.5(*m*), 839.8(*s*), 558.3(*s*).

## Refinement details   

Crystal data, data collection and structure refinement details are summarized in Table 2 ▸. H atoms bound to C atoms were placed at calculated positions [C—H = 0.99 Å (CH_2_) or 0.98 Å (CH_3_)] and refined as riding with *U*_iso_(H) = 1.2*U*_eq_(C) for CH_2_ and *U*_iso_(H) = 1.5*U*_eq_(C) for CH_3_. All H atoms bound to N atoms were refined with bond-length restraints [N—H = 0.90 (2) Å] and with *U*_iso_(H) = 1.2*U*_eq_(N). Two F atoms in one PF_6_ anion are disordered over two positions (F25*A*/F25*B* and F26*A*/F26*B*) with refined occupancies of 0.61 (4) and 0.39 (4). Two F atoms in another PF_6_ anion are also disordered over two positions (F20*A*, F21*A*, F22*A*, F23*A*) with site occupancies of 0.5. Reflections (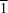 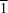 7 24) and (24 2 3) were omitted omitted owing to poor agreement between measured and calculated intensities.

## Supplementary Material

Crystal structure: contains datablock(s) I. DOI: 10.1107/S2056989017005229/is5473sup1.cif

Structure factors: contains datablock(s) I. DOI: 10.1107/S2056989017005229/is5473Isup2.hkl

CCDC reference: 1542478

Additional supporting information:  crystallographic information; 3D view; checkCIF report

## Figures and Tables

**Table 1:** Hydrogen-bond geometry (Å, °)

*D*—H⋯*A*	*D*—H	H⋯*A*	*D*⋯*A*	*D*—H⋯*A*
N1—H1⋯F15^i^	0.89 (2)	2.18 (3)	2.964 (5)	146 (4)
N1—H1⋯F17^i^	0.89 (2)	2.52 (3)	3.255 (5)	140 (4)
N2—H2⋯F3^ii^	0.89 (2)	2.55 (4)	3.204 (5)	130 (4)
N2—H2⋯F5^ii^	0.89 (2)	2.37 (3)	3.120 (5)	142 (4)
N3—H3⋯F1	0.89 (2)	2.34 (4)	3.068 (5)	139 (4)
N5—H4⋯F6	0.91 (2)	2.51 (3)	3.387 (6)	161 (5)
N7—H6⋯F11^iii^	0.90 (2)	2.28 (3)	3.158 (6)	163 (5)
C3—H11⋯F3^ii^	0.99	2.41	3.138 (5)	130
C4—H14⋯F15^i^	0.99	2.23	3.193 (5)	165
C7—H19⋯F15^i^	0.99	2.45	3.126 (6)	125
C7—H19⋯F18^iv^	0.99	2.33	3.106 (5)	134
C8—H22⋯F16^iv^	0.99	2.32	3.248 (6)	157
C8—H22⋯F18^iv^	0.99	2.55	3.267 (6)	129
C9—H24⋯F19	0.99	2.46	3.384 (7)	155
C10—H26⋯F11^v^	0.98	2.49	3.458 (6)	168
C10—H26⋯F12^v^	0.98	2.50	3.295 (6)	138
C10—H27⋯S3	0.98	2.68	3.315 (5)	123
C11—H30⋯S1^vi^	0.98	2.83	3.770 (5)	162
C14—H35⋯F18^iv^	0.99	2.27	3.091 (6)	140
C15—H38⋯F25*B* ^vii^	0.99	2.41	3.294 (16)	148
C16—H40⋯F17	0.99	2.41	3.246 (5)	142
C19—H45⋯F8^vii^	0.99	2.32	3.269 (7)	159
C19—H46⋯F24^vii^	0.99	2.44	3.420 (7)	172
C19—H46⋯F26*A* ^vii^	0.99	2.52	3.290 (13)	134
C20—H47⋯F9^iii^	0.99	2.39	3.299 (8)	152
C21—H51⋯S5	0.98	2.77	3.386 (6)	122

Symmetry codes: (i) 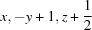; (ii) 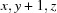; (iii) 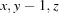; (iv) 
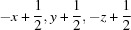
; (v) 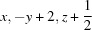; (vi) 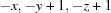; (vii) 
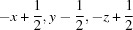
.

**Table 2:** Experimental details

Crystal data	
Chemical formula	[Co(C_11_H_27_N_4_S_3_)]·2PF_6_
*M*_r_	660.41
Crystal system, space group	Monoclinic, *C*2/*c*
Temperature (K)	200
*a*, *b*, *c* (Å)	32.440 (3), 10.3197 (8), 29.869 (2)
β (°)	110.629 (8)
*V* (Å^3^)	9358.1 (13)
*Z*	16
Radiation type	Mo *K*α
μ (mm^−1^)	1.24
Crystal size (mm)	0.15 × 0.05 × 0.05

Data collection	
Diffractometer	Rigaku R-AXIS RAPID
Absorption correction	Multi-scan (*ABSCOR*; Higashi, 1995 ▸)
*T*_min_, *T*_max_	0.776, 0.940
No. of measured, independent and observed [*I* > 2σ(*I*)] reflections	44747, 10620, 8276
*R*_int_	0.034
(sin θ/λ)_max_ (Å^−1^)	0.648

Refinement	
*R*[*F* ^2^ > 2σ(*F* ^2^)], *wR*(*F* ^2^), *S*	0.056, 0.152, 1.05
No. of reflections	10620
No. of parameters	652
No. of restraints	6
H-atom treatment	H atoms treated by a mixture of independent and constrained refinement
Δρ_max_, Δρ_min_ (e Å^−3^)	2.26, −0.66

Computer programs: *PROCESS-AUTO* (Rigaku, 2000 ▸), *SHELXS2014/7* (Sheldrick, 2008 ▸), *SHELXL2014/7* (Sheldrick, 2015 ▸) and *Mercury* (Macrae *et al.*, 2006 ▸).

## supplementary crystallographic information

### Crystal data

**Table:**

[Co(C_11_H_27_N_4_S_3_)]·2PF_6_	*F*(000) = 5344
*M**_r_* = 660.41	*D*_x_ = 1.875 Mg m^−^^3^
Monoclinic, *C*2/*c*	Mo *K*α radiation, λ = 0.71075 Å
*a* = 32.440 (3) Å	Cell parameters from 27114 reflections
*b* = 10.3197 (8) Å	θ = 3.1–27.4°
*c* = 29.869 (2) Å	µ = 1.24 mm^−^^1^
β = 110.629 (8)°	*T* = 200 K
*V* = 9358.1 (13) Å^3^	Block, purple–brown
*Z* = 16	0.15 × 0.05 × 0.05 mm

### Data collection

**Table:**

Rigaku R-AXIS RAPID diffractometer	10620 independent reflections
Radiation source: fine-focus sealed tube	8276 reflections with *I* > 2σ(*I*)
Graphite monochromator	*R*_int_ = 0.034
Detector resolution: 10.00 pixels mm^-1^	θ_max_ = 27.4°, θ_min_ = 3.1°
ω scans	*h* = −42→42
Absorption correction: multi-scan (ABSCOR; Higashi, 1995)	*k* = −13→13
*T*_min_ = 0.776, *T*_max_ = 0.940	*l* = −38→38
44747 measured reflections	

### Refinement

**Table:**

Refinement on *F*^2^	6 restraints
Least-squares matrix: full	Hydrogen site location: mixed
*R*[*F*^2^ > 2σ(*F*^2^)] = 0.056	H atoms treated by a mixture of independent and constrained refinement
*wR*(*F*^2^) = 0.152	*w* = 1/[σ^2^(*F*_o_^2^) + (0.069*P*)^2^ + 65.6905*P*] where *P* = (*F*_o_^2^ + 2*F*_c_^2^)/3
*S* = 1.05	(Δ/σ)_max_ = 0.001
10620 reflections	Δρ_max_ = 2.26 e Å^−^^3^
652 parameters	Δρ_min_ = −0.66 e Å^−^^3^

### Special details

**Table:**

Geometry. All esds (except the esd in the dihedral angle between two l.s. planes) are estimated using the full covariance matrix. The cell esds are taken into account individually in the estimation of esds in distances, angles and torsion angles; correlations between esds in cell parameters are only used when they are defined by crystal symmetry. An approximate (isotropic) treatment of cell esds is used for estimating esds involving l.s. planes.

### Fractional atomic coordinates and isotropic or equivalent isotropic displacement parameters (Å^2^)

**Table:**

	*x*	*y*	*z*	*U*_iso_*/*U*_eq_	Occ. (<1)
Co1	0.08029 (2)	0.71216 (5)	0.48620 (2)	0.01652 (12)	
Co2	0.15690 (2)	0.27337 (5)	0.27070 (2)	0.02012 (13)	
S1	0.05707 (4)	0.57397 (10)	0.53048 (4)	0.0262 (2)	
S2	0.11361 (3)	0.82080 (9)	0.55401 (3)	0.0245 (2)	
S3	0.01920 (3)	0.83717 (9)	0.46457 (4)	0.0230 (2)	
S4	0.09529 (4)	0.24869 (12)	0.20807 (4)	0.0353 (3)	
S5	0.12972 (4)	0.42822 (11)	0.30553 (5)	0.0360 (3)	
S6	0.17945 (4)	0.41336 (12)	0.22687 (4)	0.0379 (3)	
N1	0.13304 (11)	0.5972 (3)	0.49852 (12)	0.0227 (7)	
H1	0.1551 (12)	0.643 (4)	0.5189 (14)	0.027*	
N2	0.10806 (11)	0.8448 (3)	0.45360 (12)	0.0225 (7)	
H2	0.0870 (12)	0.896 (4)	0.4346 (14)	0.027*	
N3	0.05072 (12)	0.6070 (3)	0.42700 (12)	0.0239 (7)	
H3	0.0536 (16)	0.526 (2)	0.4373 (16)	0.029*	
N4	0.11984 (12)	0.6467 (3)	0.41348 (13)	0.0278 (7)	
N5	0.13157 (13)	0.1384 (4)	0.30484 (14)	0.0318 (8)	
H4	0.1273 (18)	0.186 (4)	0.3285 (14)	0.038*	
N6	0.21531 (13)	0.2955 (4)	0.32452 (13)	0.0307 (8)	
H5	0.2342 (14)	0.301 (5)	0.3088 (17)	0.037*	
N7	0.18589 (14)	0.1309 (4)	0.24699 (14)	0.0334 (8)	
H6	0.1650 (14)	0.086 (5)	0.2242 (14)	0.040*	
N8	0.20751 (15)	0.0615 (4)	0.33089 (16)	0.0419 (10)	
C1	0.10998 (15)	0.4941 (4)	0.55955 (16)	0.0316 (9)	
H7	0.1295	0.5496	0.5854	0.038*	
H8	0.1057	0.4108	0.5738	0.038*	
C2	0.13023 (15)	0.4709 (4)	0.52253 (17)	0.0302 (9)	
H9	0.1121	0.4083	0.4985	0.036*	
H10	0.1601	0.4337	0.5377	0.036*	
C3	0.12854 (15)	0.9674 (4)	0.52985 (15)	0.0290 (9)	
H11	0.1030	1.0266	0.5180	0.035*	
H12	0.1528	1.0129	0.5548	0.035*	
C4	0.14313 (14)	0.9257 (4)	0.48903 (16)	0.0273 (9)	
H13	0.1491	1.0032	0.4728	0.033*	
H14	0.1707	0.8749	0.5018	0.033*	
C5	−0.00327 (15)	0.7825 (4)	0.40281 (15)	0.0311 (9)	
H15	−0.0350	0.8041	0.3889	0.037*	
H16	0.0121	0.8260	0.3836	0.037*	
C6	0.00295 (14)	0.6384 (4)	0.40208 (16)	0.0307 (9)	
H17	−0.0149	0.5946	0.4184	0.037*	
H18	−0.0069	0.6072	0.3686	0.037*	
C7	0.14570 (16)	0.5748 (5)	0.45501 (16)	0.0324 (10)	
H19	0.1771	0.5984	0.4631	0.039*	
H20	0.1427	0.4812	0.4471	0.039*	
C8	0.12538 (17)	0.7852 (4)	0.41757 (18)	0.0346 (10)	
H21	0.1100	0.8249	0.3859	0.042*	
H22	0.1571	0.8059	0.4269	0.042*	
C9	0.07530 (16)	0.6046 (5)	0.39253 (15)	0.0314 (9)	
H23	0.0749	0.5152	0.3804	0.038*	
H24	0.0598	0.6610	0.3649	0.038*	
C10	0.07503 (17)	0.8821 (5)	0.58003 (16)	0.0362 (11)	
H25	0.0644	0.8105	0.5946	0.043*	
H26	0.0897	0.9465	0.6046	0.043*	
H27	0.0501	0.9226	0.5551	0.043*	
C11	−0.02201 (14)	0.7763 (4)	0.48704 (17)	0.0318 (9)	
H28	−0.0139	0.7994	0.5209	0.038*	
H29	−0.0506	0.8146	0.4688	0.038*	
H30	−0.0238	0.6818	0.4836	0.038*	
C12	0.06268 (17)	0.1607 (6)	0.23686 (19)	0.0447 (13)	
H31	0.0505	0.2211	0.2548	0.054*	
H32	0.0380	0.1146	0.2127	0.054*	
C13	0.09328 (17)	0.0671 (5)	0.26994 (18)	0.0377 (11)	
H33	0.1041	0.0047	0.2514	0.045*	
H34	0.0776	0.0180	0.2875	0.045*	
C14	0.17427 (18)	0.4229 (6)	0.36311 (18)	0.0435 (12)	
H35	0.1741	0.5026	0.3815	0.052*	
H36	0.1705	0.3475	0.3818	0.052*	
C15	0.21699 (17)	0.4123 (5)	0.35474 (17)	0.0377 (11)	
H37	0.2219	0.4911	0.3383	0.045*	
H38	0.2416	0.4042	0.3857	0.045*	
C16	0.1932 (2)	0.2920 (6)	0.19060 (18)	0.0471 (14)	
H39	0.1661	0.2622	0.1649	0.057*	
H40	0.2132	0.3298	0.1755	0.057*	
C17	0.21506 (18)	0.1810 (5)	0.2211 (2)	0.0440 (13)	
H41	0.2437	0.2087	0.2446	0.053*	
H42	0.2206	0.1113	0.2012	0.053*	
C18	0.16580 (19)	0.0451 (6)	0.3356 (2)	0.0495 (14)	
H43	0.1690	0.0577	0.3695	0.059*	
H44	0.1555	−0.0448	0.3266	0.059*	
C19	0.23114 (17)	0.1749 (5)	0.35411 (18)	0.0420 (12)	
H45	0.2629	0.1627	0.3601	0.050*	
H46	0.2275	0.1859	0.3854	0.050*	
C20	0.21048 (19)	0.0348 (5)	0.2865 (2)	0.0471 (13)	
H47	0.1986	−0.0532	0.2765	0.057*	
H48	0.2420	0.0342	0.2898	0.057*	
C21	0.0641 (2)	0.3959 (6)	0.1894 (2)	0.0624 (18)	
H49	0.0788	0.4520	0.1732	0.075*	
H50	0.0344	0.3750	0.1675	0.075*	
H51	0.0622	0.4409	0.2175	0.075*	
C22	0.1360 (2)	0.5917 (5)	0.2862 (2)	0.0495 (14)	
H52	0.1135	0.6080	0.2548	0.059*	
H53	0.1327	0.6542	0.3094	0.059*	
H54	0.1653	0.6011	0.2841	0.059*	
P1	0.05584 (4)	0.18660 (11)	0.38937 (4)	0.0296 (2)	
P2	0.14674 (5)	0.80321 (13)	0.16331 (5)	0.0396 (3)	
P3	0.24920 (4)	0.27568 (10)	0.09047 (4)	0.0244 (2)	
P4	0.0000	0.7599 (2)	0.2500	0.0460 (5)	
P5	0.2500	0.7500	0.0000	0.0578 (7)	
F1	0.03836 (15)	0.3159 (3)	0.40550 (14)	0.0682 (11)	
F2	0.00936 (15)	0.1243 (5)	0.38190 (19)	0.0924 (15)	
F3	0.07238 (15)	0.1369 (5)	0.44241 (12)	0.0823 (15)	
F4	0.03814 (14)	0.2313 (5)	0.33558 (12)	0.0750 (12)	
F5	0.07380 (19)	0.0568 (4)	0.37470 (16)	0.0943 (17)	
F6	0.10139 (14)	0.2515 (5)	0.39524 (18)	0.0919 (15)	
F7	0.1835 (2)	0.8970 (6)	0.1613 (2)	0.125 (2)	
F8	0.16976 (15)	0.6799 (4)	0.15072 (18)	0.0881 (14)	
F9	0.1735 (2)	0.7779 (5)	0.21836 (16)	0.117 (2)	
F10	0.10903 (15)	0.7066 (4)	0.16580 (16)	0.0770 (12)	
F11	0.12389 (16)	0.9221 (4)	0.17849 (15)	0.0790 (13)	
F12	0.11752 (15)	0.8265 (4)	0.10919 (12)	0.0712 (12)	
F13	0.21775 (9)	0.1639 (3)	0.09755 (11)	0.0408 (7)	
F14	0.27659 (10)	0.2728 (3)	0.14668 (9)	0.0409 (7)	
F15	0.22093 (10)	0.2779 (3)	0.03391 (9)	0.0415 (7)	
F16	0.28000 (9)	0.3869 (3)	0.08289 (10)	0.0363 (6)	
F17	0.21670 (9)	0.3829 (3)	0.09833 (10)	0.0390 (6)	
F18	0.28079 (10)	0.1681 (3)	0.08159 (12)	0.0437 (7)	
F19	0.04851 (15)	0.7647 (6)	0.28725 (17)	0.0966 (16)	
F20A	0.0024 (10)	0.6209 (14)	0.2713 (7)	0.190 (11)	0.5
F21A	0.0161 (5)	0.686 (2)	0.2156 (5)	0.117 (7)	0.5
F22A	0.0033 (4)	0.8942 (10)	0.2309 (4)	0.122 (7)	0.5
F23A	−0.0201 (5)	0.799 (3)	0.2890 (5)	0.142 (8)	0.5
F24	0.29174 (18)	0.6997 (7)	0.04286 (15)	0.113 (2)	
F25A	0.2683 (6)	0.8853 (11)	0.0199 (6)	0.122 (8)	0.61 (4)
F26A	0.2210 (4)	0.746 (3)	0.0318 (4)	0.114 (9)	0.61 (4)
F25B	0.2339 (10)	0.840 (4)	0.0313 (6)	0.138 (19)	0.39 (4)
F26B	0.2294 (7)	0.635 (3)	0.0177 (13)	0.133 (17)	0.39 (4)

### Atomic displacement parameters (Å^2^)

**Table:**

	*U*^11^	*U*^22^	*U*^33^	*U*^12^	*U*^13^	*U*^23^
Co1	0.0164 (3)	0.0153 (2)	0.0182 (2)	−0.00006 (18)	0.00651 (19)	0.00067 (19)
Co2	0.0229 (3)	0.0202 (3)	0.0188 (2)	0.0004 (2)	0.0093 (2)	0.0001 (2)
S1	0.0260 (5)	0.0244 (5)	0.0303 (5)	−0.0010 (4)	0.0125 (4)	0.0068 (4)
S2	0.0254 (5)	0.0221 (4)	0.0224 (4)	−0.0003 (4)	0.0042 (4)	−0.0007 (4)
S3	0.0198 (5)	0.0224 (4)	0.0268 (5)	0.0022 (4)	0.0081 (4)	0.0020 (4)
S4	0.0317 (6)	0.0391 (6)	0.0282 (5)	−0.0027 (5)	0.0020 (5)	0.0042 (5)
S5	0.0348 (6)	0.0319 (6)	0.0458 (6)	0.0000 (5)	0.0200 (5)	−0.0067 (5)
S6	0.0437 (7)	0.0372 (6)	0.0359 (6)	−0.0061 (5)	0.0182 (5)	0.0054 (5)
N1	0.0220 (18)	0.0197 (15)	0.0267 (17)	0.0016 (13)	0.0090 (14)	0.0010 (13)
N2	0.0211 (18)	0.0218 (16)	0.0267 (17)	0.0005 (13)	0.0110 (14)	0.0026 (13)
N3	0.0236 (18)	0.0239 (16)	0.0232 (16)	−0.0019 (13)	0.0071 (14)	−0.0029 (14)
N4	0.030 (2)	0.0276 (17)	0.0305 (18)	0.0009 (15)	0.0166 (15)	−0.0012 (15)
N5	0.030 (2)	0.0329 (19)	0.0300 (18)	−0.0015 (16)	0.0077 (16)	0.0029 (16)
N6	0.0240 (19)	0.040 (2)	0.0271 (18)	−0.0012 (16)	0.0078 (15)	−0.0044 (16)
N7	0.032 (2)	0.036 (2)	0.034 (2)	0.0015 (16)	0.0141 (17)	−0.0059 (17)
N8	0.041 (2)	0.037 (2)	0.045 (2)	0.0102 (18)	0.011 (2)	0.0131 (19)
C1	0.032 (2)	0.028 (2)	0.032 (2)	0.0014 (18)	0.0067 (18)	0.0118 (18)
C2	0.027 (2)	0.0194 (18)	0.042 (2)	0.0065 (16)	0.0090 (19)	0.0071 (18)
C3	0.032 (2)	0.0187 (18)	0.030 (2)	−0.0036 (16)	0.0036 (18)	0.0004 (16)
C4	0.022 (2)	0.0233 (19)	0.036 (2)	−0.0044 (16)	0.0086 (17)	0.0028 (17)
C5	0.026 (2)	0.038 (2)	0.025 (2)	0.0008 (18)	0.0026 (17)	0.0028 (18)
C6	0.021 (2)	0.038 (2)	0.028 (2)	−0.0065 (18)	0.0035 (17)	−0.0067 (18)
C7	0.034 (3)	0.034 (2)	0.034 (2)	0.0100 (19)	0.019 (2)	−0.0007 (19)
C8	0.045 (3)	0.029 (2)	0.043 (3)	−0.0027 (19)	0.032 (2)	−0.001 (2)
C9	0.035 (3)	0.037 (2)	0.025 (2)	−0.0017 (19)	0.0144 (18)	−0.0048 (18)
C10	0.046 (3)	0.038 (2)	0.028 (2)	0.000 (2)	0.017 (2)	−0.0111 (19)
C11	0.021 (2)	0.036 (2)	0.042 (2)	0.0013 (17)	0.0161 (19)	0.002 (2)
C12	0.027 (3)	0.053 (3)	0.045 (3)	−0.010 (2)	0.001 (2)	0.010 (2)
C13	0.040 (3)	0.035 (2)	0.040 (3)	−0.011 (2)	0.017 (2)	0.001 (2)
C14	0.044 (3)	0.056 (3)	0.034 (2)	−0.010 (2)	0.019 (2)	−0.018 (2)
C15	0.038 (3)	0.045 (3)	0.029 (2)	−0.011 (2)	0.010 (2)	−0.015 (2)
C16	0.059 (4)	0.060 (3)	0.035 (2)	−0.022 (3)	0.032 (3)	−0.009 (2)
C17	0.039 (3)	0.053 (3)	0.052 (3)	−0.006 (2)	0.030 (2)	−0.020 (3)
C18	0.044 (3)	0.044 (3)	0.051 (3)	0.001 (2)	0.007 (3)	0.027 (3)
C19	0.033 (3)	0.052 (3)	0.034 (2)	0.004 (2)	0.004 (2)	0.007 (2)
C20	0.045 (3)	0.039 (3)	0.055 (3)	0.020 (2)	0.015 (3)	0.004 (2)
C21	0.047 (4)	0.047 (3)	0.068 (4)	0.005 (3)	−0.012 (3)	0.021 (3)
C22	0.058 (4)	0.023 (2)	0.069 (4)	0.002 (2)	0.023 (3)	−0.006 (2)
P1	0.0333 (6)	0.0279 (5)	0.0304 (6)	0.0035 (5)	0.0148 (5)	0.0061 (4)
P2	0.0383 (7)	0.0350 (6)	0.0364 (6)	0.0008 (5)	0.0018 (5)	−0.0048 (5)
P3	0.0188 (5)	0.0282 (5)	0.0262 (5)	0.0015 (4)	0.0081 (4)	−0.0010 (4)
P4	0.0453 (12)	0.0578 (12)	0.0330 (9)	0.000	0.0114 (8)	0.000
P5	0.0677 (15)	0.0786 (16)	0.0216 (8)	−0.0354 (13)	0.0088 (9)	−0.0055 (9)
F1	0.094 (3)	0.0412 (18)	0.076 (2)	0.0213 (19)	0.038 (2)	−0.0003 (17)
F2	0.070 (3)	0.097 (3)	0.117 (4)	−0.040 (3)	0.042 (3)	−0.012 (3)
F3	0.105 (3)	0.111 (3)	0.0423 (18)	0.067 (3)	0.040 (2)	0.037 (2)
F4	0.078 (3)	0.101 (3)	0.0385 (18)	0.022 (2)	0.0118 (18)	0.0202 (19)
F5	0.169 (5)	0.060 (2)	0.082 (3)	0.054 (3)	0.080 (3)	0.011 (2)
F6	0.041 (2)	0.134 (4)	0.099 (3)	−0.028 (3)	0.024 (2)	−0.005 (3)
F7	0.107 (4)	0.113 (4)	0.171 (6)	−0.078 (4)	0.069 (4)	−0.036 (4)
F8	0.071 (3)	0.070 (3)	0.117 (4)	0.012 (2)	0.025 (3)	−0.025 (3)
F9	0.148 (5)	0.084 (3)	0.058 (3)	0.020 (3)	−0.041 (3)	−0.005 (2)
F10	0.081 (3)	0.068 (2)	0.081 (3)	−0.019 (2)	0.028 (2)	0.015 (2)
F11	0.101 (3)	0.061 (2)	0.070 (2)	0.025 (2)	0.023 (2)	−0.016 (2)
F12	0.108 (3)	0.054 (2)	0.0348 (17)	−0.008 (2)	0.0039 (19)	0.0041 (15)
F13	0.0325 (15)	0.0400 (15)	0.0523 (17)	−0.0091 (12)	0.0179 (13)	0.0015 (13)
F14	0.0392 (16)	0.0475 (16)	0.0278 (13)	0.0010 (13)	0.0014 (12)	0.0038 (12)
F15	0.0383 (16)	0.0545 (18)	0.0263 (13)	−0.0067 (13)	0.0048 (12)	−0.0036 (12)
F16	0.0340 (15)	0.0340 (14)	0.0439 (15)	−0.0083 (11)	0.0174 (12)	−0.0021 (12)
F17	0.0371 (16)	0.0403 (15)	0.0459 (15)	0.0168 (12)	0.0223 (13)	0.0058 (13)
F18	0.0402 (17)	0.0351 (14)	0.0628 (19)	0.0093 (12)	0.0269 (15)	−0.0031 (14)
F19	0.060 (3)	0.139 (4)	0.073 (3)	0.005 (3)	0.000 (2)	0.022 (3)
F20A	0.37 (3)	0.069 (8)	0.131 (15)	−0.062 (13)	0.08 (2)	0.014 (7)
F21A	0.114 (12)	0.180 (19)	0.058 (8)	0.076 (14)	0.030 (7)	−0.015 (11)
F22A	0.079 (8)	0.082 (6)	0.148 (14)	−0.029 (7)	−0.030 (11)	0.069 (7)
F23A	0.098 (10)	0.28 (2)	0.062 (7)	−0.009 (15)	0.049 (7)	−0.045 (13)
F24	0.093 (4)	0.191 (6)	0.041 (2)	0.009 (4)	0.007 (2)	0.009 (3)
F25A	0.169 (14)	0.088 (7)	0.088 (10)	−0.061 (7)	0.019 (9)	−0.029 (6)
F26A	0.083 (7)	0.22 (3)	0.050 (5)	−0.032 (11)	0.032 (5)	0.015 (9)
F25B	0.13 (3)	0.22 (3)	0.042 (8)	0.05 (3)	0.007 (10)	−0.053 (15)
F26B	0.122 (15)	0.12 (2)	0.16 (3)	−0.037 (13)	0.051 (15)	0.05 (2)

### Geometric parameters (Å, º)

**Table:**

Co1—N1	2.007 (3)	C10—H26	0.9800
Co1—N3	2.007 (3)	C10—H27	0.9800
Co1—N2	2.062 (3)	C11—H28	0.9800
Co1—S2	2.2327 (11)	C11—H29	0.9800
Co1—S1	2.2478 (11)	C11—H30	0.9800
Co1—S3	2.2599 (11)	C12—C13	1.484 (7)
Co2—N7	2.004 (4)	C12—H31	0.9900
Co2—N6	2.020 (4)	C12—H32	0.9900
Co2—N5	2.061 (4)	C13—H33	0.9900
Co2—S4	2.2190 (13)	C13—H34	0.9900
Co2—S6	2.2384 (13)	C14—C15	1.496 (7)
Co2—S5	2.2494 (12)	C14—H35	0.9900
S1—C1	1.825 (5)	C14—H36	0.9900
S2—C10	1.805 (5)	C15—H37	0.9900
S2—C3	1.814 (4)	C15—H38	0.9900
S3—C11	1.807 (4)	C16—C17	1.480 (8)
S3—C5	1.818 (4)	C16—H39	0.9900
S4—C21	1.801 (6)	C16—H40	0.9900
S4—C12	1.823 (5)	C17—H41	0.9900
S5—C14	1.816 (5)	C17—H42	0.9900
S5—C22	1.818 (5)	C18—H43	0.9900
S6—C16	1.812 (5)	C18—H44	0.9900
N1—C2	1.506 (5)	C19—H45	0.9900
N1—C7	1.512 (5)	C19—H46	0.9900
N1—H1	0.893 (19)	C20—H47	0.9900
N2—C4	1.504 (5)	C20—H48	0.9900
N2—C8	1.509 (5)	C21—H49	0.9800
N2—H2	0.893 (19)	C21—H50	0.9800
N3—C6	1.499 (6)	C21—H51	0.9800
N3—C9	1.508 (5)	C22—H52	0.9800
N3—H3	0.889 (19)	C22—H53	0.9800
N4—C9	1.425 (6)	C22—H54	0.9800
N4—C7	1.435 (6)	P1—F3	1.569 (3)
N4—C8	1.441 (6)	P1—F4	1.573 (3)
N5—C13	1.503 (6)	P1—F6	1.575 (4)
N5—C18	1.512 (6)	P1—F2	1.581 (4)
N5—H4	0.913 (19)	P1—F5	1.583 (4)
N6—C15	1.496 (6)	P1—F1	1.590 (3)
N6—C19	1.508 (6)	P2—F7	1.554 (4)
N6—H5	0.895 (19)	P2—F12	1.578 (4)
N7—C17	1.508 (6)	P2—F11	1.580 (4)
N7—C20	1.533 (6)	P2—F8	1.587 (4)
N7—H6	0.904 (19)	P2—F9	1.587 (4)
N8—C20	1.391 (7)	P2—F10	1.600 (4)
N8—C18	1.419 (7)	P3—F16	1.589 (3)
N8—C19	1.436 (7)	P3—F18	1.595 (3)
C1—C2	1.491 (6)	P3—F14	1.599 (3)
C1—H7	0.9900	P3—F17	1.602 (3)
C1—H8	0.9900	P3—F13	1.603 (3)
C2—H9	0.9900	P3—F15	1.614 (3)
C2—H10	0.9900	P4—F21A	1.515 (11)
C3—C4	1.517 (6)	P4—F21A^i^	1.515 (11)
C3—H11	0.9900	P4—F22A	1.516 (9)
C3—H12	0.9900	P4—F20A	1.560 (15)
C4—H13	0.9900	P4—F23A	1.575 (11)
C4—H14	0.9900	P4—F19^i^	1.576 (4)
C5—C6	1.501 (6)	P4—F19	1.576 (4)
C5—H15	0.9900	P5—F25B^ii^	1.535 (17)
C5—H16	0.9900	P5—F25B	1.535 (17)
C6—H17	0.9900	P5—F26B	1.543 (14)
C6—H18	0.9900	P5—F26B^ii^	1.544 (14)
C7—H19	0.9900	P5—F25A	1.551 (9)
C7—H20	0.9900	P5—F25A^ii^	1.551 (9)
C8—H21	0.9900	P5—F26A	1.556 (11)
C8—H22	0.9900	P5—F26A^ii^	1.557 (11)
C9—H23	0.9900	P5—F24	1.588 (5)
C9—H24	0.9900	P5—F24^ii^	1.588 (5)
C10—H25	0.9800		
			
N1—Co1—N3	87.36 (14)	S4—C12—H32	110.5
N1—Co1—N2	89.58 (14)	H31—C12—H32	108.7
N3—Co1—N2	95.48 (14)	C12—C13—N5	109.8 (4)
N1—Co1—S2	91.23 (10)	C12—C13—H33	109.7
N3—Co1—S2	177.38 (11)	N5—C13—H33	109.7
N2—Co1—S2	86.72 (10)	C12—C13—H34	109.7
N1—Co1—S1	87.59 (10)	N5—C13—H34	109.7
N3—Co1—S1	91.15 (10)	H33—C13—H34	108.2
N2—Co1—S1	172.66 (10)	C15—C14—S5	108.6 (3)
S2—Co1—S1	86.58 (4)	C15—C14—H35	110.0
N1—Co1—S3	174.34 (10)	S5—C14—H35	110.0
N3—Co1—S3	87.59 (11)	C15—C14—H36	110.0
N2—Co1—S3	88.35 (10)	S5—C14—H36	110.0
S2—Co1—S3	93.91 (4)	H35—C14—H36	108.4
S1—Co1—S3	95.07 (4)	N6—C15—C14	108.7 (4)
N7—Co2—N6	86.49 (16)	N6—C15—H37	109.9
N7—Co2—N5	89.86 (16)	C14—C15—H37	109.9
N6—Co2—N5	96.21 (16)	N6—C15—H38	109.9
N7—Co2—S4	90.77 (13)	C14—C15—H38	109.9
N6—Co2—S4	176.01 (11)	H37—C15—H38	108.3
N5—Co2—S4	86.67 (11)	C17—C16—S6	109.4 (3)
N7—Co2—S6	88.63 (12)	C17—C16—H39	109.8
N6—Co2—S6	89.11 (12)	S6—C16—H39	109.8
N5—Co2—S6	174.37 (12)	C17—C16—H40	109.8
S4—Co2—S6	87.93 (5)	S6—C16—H40	109.8
N7—Co2—S5	173.60 (13)	H39—C16—H40	108.2
N6—Co2—S5	87.84 (12)	C16—C17—N7	109.3 (4)
N5—Co2—S5	87.81 (12)	C16—C17—H41	109.8
S4—Co2—S5	95.05 (5)	N7—C17—H41	109.8
S6—Co2—S5	94.25 (5)	C16—C17—H42	109.8
C1—S1—Co1	96.47 (15)	N7—C17—H42	109.8
C10—S2—C3	101.6 (2)	H41—C17—H42	108.3
C10—S2—Co1	112.28 (16)	N8—C18—N5	112.7 (4)
C3—S2—Co1	99.77 (14)	N8—C18—H43	109.1
C11—S3—C5	100.6 (2)	N5—C18—H43	109.1
C11—S3—Co1	112.61 (15)	N8—C18—H44	109.1
C5—S3—Co1	96.35 (15)	N5—C18—H44	109.1
C21—S4—C12	102.1 (3)	H43—C18—H44	107.8
C21—S4—Co2	113.9 (2)	N8—C19—N6	112.1 (4)
C12—S4—Co2	99.18 (17)	N8—C19—H45	109.2
C14—S5—C22	100.8 (3)	N6—C19—H45	109.2
C14—S5—Co2	96.08 (18)	N8—C19—H46	109.2
C22—S5—Co2	114.0 (2)	N6—C19—H46	109.2
C16—S6—Co2	95.95 (17)	H45—C19—H46	107.9
C2—N1—C7	110.9 (3)	N8—C20—N7	114.4 (4)
C2—N1—Co1	114.1 (3)	N8—C20—H47	108.7
C7—N1—Co1	113.9 (3)	N7—C20—H47	108.7
C2—N1—H1	107 (3)	N8—C20—H48	108.7
C7—N1—H1	106 (3)	N7—C20—H48	108.7
Co1—N1—H1	104 (3)	H47—C20—H48	107.6
C4—N2—C8	110.4 (3)	S4—C21—H49	109.5
C4—N2—Co1	112.6 (2)	S4—C21—H50	109.5
C8—N2—Co1	113.6 (3)	H49—C21—H50	109.5
C4—N2—H2	110 (3)	S4—C21—H51	109.5
C8—N2—H2	101 (3)	H49—C21—H51	109.5
Co1—N2—H2	109 (3)	H50—C21—H51	109.5
C6—N3—C9	111.6 (3)	S5—C22—H52	109.5
C6—N3—Co1	114.2 (3)	S5—C22—H53	109.5
C9—N3—Co1	114.0 (3)	H52—C22—H53	109.5
C6—N3—H3	110 (3)	S5—C22—H54	109.5
C9—N3—H3	102 (3)	H52—C22—H54	109.5
Co1—N3—H3	104 (3)	H53—C22—H54	109.5
C9—N4—C7	114.5 (4)	F3—P1—F4	177.7 (3)
C9—N4—C8	114.6 (4)	F3—P1—F6	93.0 (3)
C7—N4—C8	115.0 (4)	F4—P1—F6	88.8 (3)
C13—N5—C18	111.1 (4)	F3—P1—F2	88.8 (3)
C13—N5—Co2	111.3 (3)	F4—P1—F2	89.3 (3)
C18—N5—Co2	113.3 (3)	F6—P1—F2	178.1 (3)
C13—N5—H4	119 (4)	F3—P1—F5	88.5 (2)
C18—N5—H4	99 (3)	F4—P1—F5	90.2 (2)
Co2—N5—H4	102 (3)	F6—P1—F5	88.2 (3)
C15—N6—C19	112.3 (4)	F2—P1—F5	92.4 (3)
C15—N6—Co2	113.3 (3)	F3—P1—F1	90.0 (2)
C19—N6—Co2	113.8 (3)	F4—P1—F1	91.3 (2)
C15—N6—H5	113 (3)	F6—P1—F1	91.7 (3)
C19—N6—H5	102 (3)	F2—P1—F1	87.7 (3)
Co2—N6—H5	102 (3)	F5—P1—F1	178.5 (2)
C17—N7—C20	111.4 (4)	F7—P2—F12	93.3 (3)
C17—N7—Co2	112.8 (3)	F7—P2—F11	88.4 (3)
C20—N7—Co2	112.6 (3)	F12—P2—F11	90.1 (2)
C17—N7—H6	103 (3)	F7—P2—F8	93.3 (3)
C20—N7—H6	107 (4)	F12—P2—F8	92.4 (2)
Co2—N7—H6	109 (4)	F11—P2—F8	176.8 (3)
C20—N8—C18	117.3 (5)	F7—P2—F9	89.8 (4)
C20—N8—C19	114.7 (5)	F12—P2—F9	176.5 (3)
C18—N8—C19	114.5 (5)	F11—P2—F9	88.3 (3)
C2—C1—S1	107.8 (3)	F8—P2—F9	89.0 (3)
C2—C1—H7	110.1	F7—P2—F10	179.6 (4)
S1—C1—H7	110.1	F12—P2—F10	87.1 (2)
C2—C1—H8	110.1	F11—P2—F10	91.6 (3)
S1—C1—H8	110.1	F8—P2—F10	86.7 (3)
H7—C1—H8	108.5	F9—P2—F10	89.8 (3)
C1—C2—N1	109.3 (3)	F16—P3—F18	90.34 (16)
C1—C2—H9	109.8	F16—P3—F14	91.06 (16)
N1—C2—H9	109.8	F18—P3—F14	90.56 (17)
C1—C2—H10	109.8	F16—P3—F17	90.09 (15)
N1—C2—H10	109.8	F18—P3—F17	178.79 (19)
H9—C2—H10	108.3	F14—P3—F17	90.57 (16)
C4—C3—S2	106.6 (3)	F16—P3—F13	179.37 (19)
C4—C3—H11	110.4	F18—P3—F13	89.83 (16)
S2—C3—H11	110.4	F14—P3—F13	89.55 (16)
C4—C3—H12	110.4	F17—P3—F13	89.74 (16)
S2—C3—H12	110.4	F16—P3—F15	89.69 (16)
H11—C3—H12	108.6	F18—P3—F15	89.84 (17)
N2—C4—C3	110.5 (3)	F14—P3—F15	179.15 (18)
N2—C4—H13	109.6	F17—P3—F15	89.03 (16)
C3—C4—H13	109.6	F13—P3—F15	89.70 (16)
N2—C4—H14	109.6	F21A—P4—F21A^i^	119.1 (19)
C3—C4—H14	109.6	F21A—P4—F22A	97.0 (9)
H13—C4—H14	108.1	F21A^i^—P4—F22A	143.5 (13)
C6—C5—S3	108.3 (3)	F21A—P4—F20A	80.3 (15)
C6—C5—H15	110.0	F22A—P4—F20A	173.6 (13)
S3—C5—H15	110.0	F21A—P4—F23A	164.4 (19)
C6—C5—H16	110.0	F21A^i^—P4—F23A	45.4 (8)
S3—C5—H16	110.0	F22A—P4—F23A	98.3 (15)
H15—C5—H16	108.4	F20A—P4—F23A	84.8 (11)
N3—C6—C5	109.1 (3)	F21A—P4—F19^i^	91.9 (6)
N3—C6—H17	109.9	F21A^i^—P4—F19^i^	89.9 (6)
C5—C6—H17	109.9	F22A—P4—F19^i^	83.7 (5)
N3—C6—H18	109.9	F20A—P4—F19^i^	102.1 (11)
C5—C6—H18	109.9	F23A—P4—F19^i^	86.7 (6)
H17—C6—H18	108.3	F21A—P4—F19	89.9 (6)
N4—C7—N1	114.2 (3)	F21A^i^—P4—F19	91.9 (6)
N4—C7—H19	108.7	F22A—P4—F19	93.0 (5)
N1—C7—H19	108.7	F20A—P4—F19	81.3 (11)
N4—C7—H20	108.7	F23A—P4—F19	92.3 (6)
N1—C7—H20	108.7	F19^i^—P4—F19	176.4 (5)
H19—C7—H20	107.6	F25B^ii^—P5—F25B	180.0 (9)
N4—C8—N2	113.4 (3)	F25B^ii^—P5—F26B	91.2 (13)
N4—C8—H21	108.9	F25B—P5—F26B	88.8 (13)
N2—C8—H21	108.9	F25B^ii^—P5—F26B^ii^	88.8 (13)
N4—C8—H22	108.9	F25B—P5—F26B^ii^	91.2 (13)
N2—C8—H22	108.9	F26B—P5—F26B^ii^	180.0
H21—C8—H22	107.7	F25A—P5—F25A^ii^	180.0
N4—C9—N3	113.2 (3)	F25A—P5—F26A	91.3 (7)
N4—C9—H23	108.9	F25A^ii^—P5—F26A	88.7 (7)
N3—C9—H23	108.9	F25A—P5—F26A^ii^	88.7 (7)
N4—C9—H24	108.9	F25A^ii^—P5—F26A^ii^	91.3 (7)
N3—C9—H24	108.9	F26A—P5—F26A^ii^	180.0
H23—C9—H24	107.8	F25B^ii^—P5—F24	85.2 (7)
S2—C10—H25	109.5	F25B—P5—F24	94.8 (7)
S2—C10—H26	109.5	F26B—P5—F24	79.7 (9)
H25—C10—H26	109.5	F26B^ii^—P5—F24	100.3 (9)
S2—C10—H27	109.5	F25A—P5—F24	83.3 (6)
H25—C10—H27	109.5	F25A^ii^—P5—F24	96.7 (6)
H26—C10—H27	109.5	F26A—P5—F24	91.5 (6)
S3—C11—H28	109.5	F26A^ii^—P5—F24	88.5 (6)
S3—C11—H29	109.5	F25B^ii^—P5—F24^ii^	94.8 (7)
H28—C11—H29	109.5	F25B—P5—F24^ii^	85.2 (7)
S3—C11—H30	109.5	F26B—P5—F24^ii^	100.3 (9)
H28—C11—H30	109.5	F26B^ii^—P5—F24^ii^	79.7 (9)
H29—C11—H30	109.5	F25A—P5—F24^ii^	96.7 (6)
C13—C12—S4	105.9 (4)	F25A^ii^—P5—F24^ii^	83.3 (6)
C13—C12—H31	110.5	F26A—P5—F24^ii^	88.5 (6)
S4—C12—H31	110.5	F26A^ii^—P5—F24^ii^	91.5 (6)
C13—C12—H32	110.5	F24—P5—F24^ii^	180.0
			
Co1—S1—C1—C2	−42.7 (3)	C21—S4—C12—C13	−157.4 (4)
S1—C1—C2—N1	55.1 (4)	Co2—S4—C12—C13	−40.4 (4)
C7—N1—C2—C1	−169.8 (4)	S4—C12—C13—N5	57.6 (5)
Co1—N1—C2—C1	−39.6 (4)	C18—N5—C13—C12	−173.9 (4)
C10—S2—C3—C4	−154.6 (3)	Co2—N5—C13—C12	−46.5 (5)
Co1—S2—C3—C4	−39.2 (3)	C22—S5—C14—C15	−73.8 (4)
C8—N2—C4—C3	−170.7 (3)	Co2—S5—C14—C15	42.0 (4)
Co1—N2—C4—C3	−42.5 (4)	C19—N6—C15—C14	−89.0 (5)
S2—C3—C4—N2	54.2 (4)	Co2—N6—C15—C14	41.6 (5)
C11—S3—C5—C6	−72.8 (3)	S5—C14—C15—N6	−56.3 (5)
Co1—S3—C5—C6	41.7 (3)	Co2—S6—C16—C17	−40.8 (4)
C9—N3—C6—C5	−90.8 (4)	S6—C16—C17—N7	55.0 (5)
Co1—N3—C6—C5	40.3 (4)	C20—N7—C17—C16	−168.5 (4)
S3—C5—C6—N3	−55.0 (4)	Co2—N7—C17—C16	−40.7 (5)
C9—N4—C7—N1	−69.8 (5)	C20—N8—C18—N5	−65.0 (6)
C8—N4—C7—N1	66.1 (5)	C19—N8—C18—N5	73.9 (6)
C2—N1—C7—N4	134.6 (4)	C13—N5—C18—N8	120.4 (5)
Co1—N1—C7—N4	4.2 (5)	Co2—N5—C18—N8	−5.9 (6)
C9—N4—C8—N2	68.7 (5)	C20—N8—C19—N6	59.4 (6)
C7—N4—C8—N2	−67.2 (5)	C18—N8—C19—N6	−80.5 (5)
C4—N2—C8—N4	126.6 (4)	C15—N6—C19—N8	145.3 (4)
Co1—N2—C8—N4	−1.0 (5)	Co2—N6—C19—N8	14.9 (5)
C7—N4—C9—N3	56.9 (5)	C18—N8—C20—N7	68.8 (6)
C8—N4—C9—N3	−79.1 (5)	C19—N8—C20—N7	−69.9 (6)
C6—N3—C9—N4	147.1 (4)	C17—N7—C20—N8	129.2 (5)
Co1—N3—C9—N4	16.0 (5)	Co2—N7—C20—N8	1.3 (6)

Symmetry codes: (i) −*x*, *y*, −*z*+1/2; (ii) −*x*+1/2, −*y*+3/2, −*z*.

### Hydrogen-bond geometry (Å, º)

**Table:**

*D*—H···*A*	*D*—H	H···*A*	*D*···*A*	*D*—H···*A*
N1—H1···F15^iii^	0.89 (2)	2.18 (3)	2.964 (5)	146 (4)
N1—H1···F17^iii^	0.89 (2)	2.52 (3)	3.255 (5)	140 (4)
N2—H2···F3^iv^	0.89 (2)	2.55 (4)	3.204 (5)	130 (4)
N2—H2···F5^iv^	0.89 (2)	2.37 (3)	3.120 (5)	142 (4)
N3—H3···F1	0.89 (2)	2.34 (4)	3.068 (5)	139 (4)
N5—H4···F6	0.91 (2)	2.51 (3)	3.387 (6)	161 (5)
N7—H6···F11^v^	0.90 (2)	2.28 (3)	3.158 (6)	163 (5)
C3—H11···F3^iv^	0.99	2.41	3.138 (5)	130
C4—H14···F15^iii^	0.99	2.23	3.193 (5)	165
C7—H19···F15^iii^	0.99	2.45	3.126 (6)	125
C7—H19···F18^vi^	0.99	2.33	3.106 (5)	134
C8—H22···F16^vi^	0.99	2.32	3.248 (6)	157
C8—H22···F18^vi^	0.99	2.55	3.267 (6)	129
C9—H24···F19	0.99	2.46	3.384 (7)	155
C10—H26···F11^vii^	0.98	2.49	3.458 (6)	168
C10—H26···F12^vii^	0.98	2.50	3.295 (6)	138
C10—H27···S3	0.98	2.68	3.315 (5)	123
C11—H30···S1^viii^	0.98	2.83	3.770 (5)	162
C14—H35···F18^vi^	0.99	2.27	3.091 (6)	140
C15—H38···F25*B*^ix^	0.99	2.41	3.294 (16)	148
C16—H40···F17	0.99	2.41	3.246 (5)	142
C19—H45···F8^ix^	0.99	2.32	3.269 (7)	159
C19—H46···F24^ix^	0.99	2.44	3.420 (7)	172
C19—H46···F26*A*^ix^	0.99	2.52	3.290 (13)	134
C20—H47···F9^v^	0.99	2.39	3.299 (8)	152
C21—H51···S5	0.98	2.77	3.386 (6)	122

Symmetry codes: (iii) *x*, −*y*+1, *z*+1/2; (iv) *x*, *y*+1, *z*; (v) *x*, *y*−1, *z*; (vi) −*x*+1/2, *y*+1/2, −*z*+1/2; (vii) *x*, −*y*+2, *z*+1/2; (viii) −*x*, −*y*+1, −*z*+1; (ix) −*x*+1/2, *y*−1/2, −*z*+1/2.
